# Antibiotic-Induced Changes in Microbiome-Related Metabolites and Bile Acids in Rat Plasma

**DOI:** 10.3390/metabo10060242

**Published:** 2020-06-11

**Authors:** Véronique de Bruijn, Christina Behr, Saskia Sperber, Tilmann Walk, Philipp Ternes, Markus Slopianka, Volker Haake, Karsten Beekmann, Bennard van Ravenzwaay

**Affiliations:** 1 BASF SE, Experimental Toxicology and Ecology, 67056 Ludwigshafen, Germany; veronique.debruijn@wur.nl (V.d.B.); NinaBehr1@gmx.de (C.B.); saskia.sperber@basf.com (S.S.); 2 Division of Toxicology, Wageningen University and Research, 6708 WE Wageningen, The Netherlands; karsten.beekmann@wur.nl; 3 BASF Metabolome Solutions, Tegeler Weg 33, 10589 Berlin, Germany; tilmann.walk@basf.com (T.W.); philipp.ternes@basf.com (P.T.); markus.slopianka@basf.com (M.S.); volker.haake@basf.com (V.H.)

**Keywords:** microbiome, bile acids, metabolomics, antibiotics

## Abstract

Various environmental factors can alter the gut microbiome’s composition and functionality, and modulate host health. In this study, the effects of oral and parenteral administration of two poorly bioavailable antibiotics (i.e., vancomycin and streptomycin) on male Wistar Crl/Wi(Han) rats for 28 days were compared to distinguish between microbiome-derived or -associated and systemic changes in the plasma metabolome. The resulting changes in the plasma metabolome were compared to the effects of a third reference compound, roxithromycin, which is readily bioavailable. A community analysis revealed that the oral administration of vancomycin and roxithromycin in particular leads to an altered microbial population. Antibiotic-induced changes depending on the administration routes were observed in plasma metabolite levels. Indole-3-acetic acid (IAA) and hippuric acid (HA) were identified as key metabolites of microbiome modulation, with HA being the most sensitive. Even though large variations in the plasma bile acid pool between and within rats were observed, the change in microbiome community was observed to alter the composition of the bile acid pool, especially by an accumulation of taurine-conjugated primary bile acids. In-depth investigation of the relationship between microbiome variability and their functionality, with emphasis on the bile acid pool, will be necessary to better assess the potential adverseness of environmentally induced microbiome changes.

## 1. Introduction

The gut microbiome consists of around 1000 species of bacteria, fungi, archaea and viruses [1] and is increasingly recognized to play a central role in host health. Co-evolution of the host with the gut microbiome has resulted in a close host-microbiome interplay and mutual co-dependency. The host body is crucial for the survival of the gut microbiome, whereas the microbiome plays a role in many metabolic processes required for host health and well-being [2].

Environmental factors can affect the composition and functionality of the microbiome. The gut microbiome is comprised of bacterial, archaeal, viral and fungal species. Alterations in the microbial composition can alter the microbiome’s functionality, i.e., the metabolites produced by the microbiome which are subsequently absorbed by the host, and metabolomics can be used to assess these changes [3]. Within the scope of metabolomics, metabolites are defined as small (<1500 Dalton) endogenous compounds such as carbohydrates, amino acids, nucleic acids or fatty acids and their derivatives resulting from biochemical pathways [4]. Sensitive mass spectrometry (MS) techniques allow for the detection of a broad range of metabolites, which is important for increasing the probability of detecting relevant biomarkers. Metabolomics can be used in the field of toxicology and pharmacology, as it allows for the rapid identification of alterations in biochemical pathways after the administration of xenobiotics. Thereby, metabolomics can provide information on likely target organs and the mode-of-action of a compound [5].

One of the key functions of the microbiome is the regulation of the bile acid pool. Bile acids aid the absorption of fats and fat-soluble vitamins from food. In the host, bile acids also regulate genes involved in lipid and glucose metabolism, and energy homeostasis via binding to numerous nuclear receptors, e.g., the trans-membrane-bound G-protein-coupled receptor (TGR5), the vitamin D receptor (VDR) and the farnesoid X receptor (FXR) [6]. Primary bile acids are predominantly synthesized in the liver from cholesterol via the classical pathway. The first and rate-limiting step in the classical pathway is the conversion of cholesterol into 7α-HO-cholesterol by the enzyme cholesterol-7α hydroxylase (CYP7A1); it is next converted into 7α-HO-4-cholesten-3-one (C4). Sterol 12-hydroxylase (CYP8B1) is required to synthesize cholic acid (CA) from C4; when 12α-hydroxylation by CYP8B1 does not take place chenodeoxycholic acid (CDCA) is synthesized. Mitochondrial steroid 27-hydroxylase (CYP27A1) catalyzes the oxidation of the steroid side-chain, followed by oxidative cleavage of a three-carbon side-chain to form C24 cholestonic acid, the backbone of most bile acids. In the alternative pathway, CYP27A1 initiates the formation of bile acids and different intermediates are formed, i.e., 3β-HO-5-cholestenoic acid and 3β,7α-(HO)_2_-5-cholestenoic acid. As well as in the liver, the alternative pathway is expressed in macrophages and steroidogenic tissues, such as the adrenal gland, ovary and testis, and contributes up to 10% to the total bile acid synthesis. More minor pathways contribute to the formation of bile acids. In the rodent liver, CDCA can be converted into α-muricholic acid (α-MCA) which is then epimerized to β-muricholic acid (β-MCA) [7,8,9]. Prior to their secretion into the small intestine, primary bile acids in rodents (i.e., CA, CDCA, α- and β-MCA) are conjugated with either taurine or glycine to give them their amphiphilic character. After secretion into the small intestine, bile acids are deconjugated by several Gram-positive bacterial species. Approximately 95% of the bile acids are actively reabsorbed in the ileum and are transported to the liver via the portal vein. In the liver, bile acids are reconjugated and subsequently secreted into the bile canaliculus and intestine again via enterohepatic circulation. The remaining bile acids that escape reabsorption enter the colon, are further metabolized by gut microbial species and are eventually partly excreted via the feces and urine [10,11]. After deconjugation, additional microbial modifications, including oxidation and dehydroxylation, occur in the colon. These enzymatic reactions give rise to unconjugated bile acids as well as secondary bile acids, such as (iso-)deoxycholic acid (DCA), tauro- and glycodeoxycholic acid (TDCA, GDCA), ω-muricholic acid (ω-MCA), hyodeoxycholic acid (HDCA), ursodeoxycholic acid (UDCA), tauro- and glycoursodeoxycholic acid (TUDCA, GUDCA), (iso-)lithocholic acid (LCA), taurolitocholic acid (TLCA) and glycolithocholic acid (GLCA) [7,10]. Perturbations of the bile acid pool are associated with several disease states, such as metabolic syndrome, non-alcoholic fatty liver disease, inflammatory bowel disease and even colorectal cancers [12,13]. Hence, it is very relevant to elucidate the impact of xenobiotic-induced modulations of the gut microbial community on the bile acid pool for a better understanding of the onset and progression of disease as well as the development of new therapeutics.

The interplay between microbial and mammalian metabolism is reflected in the plasma metabolome [3,14,15,16]. Previously, glycerol, indole-derivatives, hippuric acid and bile acids were identified as key metabolites altered by the microbiome [3,14]. The current project uses a novel approach to identify microbiome-derived metabolites and to distinguish these from those that arise from systemic toxicity. To this end, male rats were administered antibiotics orally or parenterally for 28 days and the antibiotic-induced effects of both administration routes on the plasma metabolome were compared. The rats were administered the orally non-bioavailable glycopeptide antibiotic vancomycin and the poorly bioavailable aminoglycoside antibiotic streptomycin to exclude or minimize systemic toxicity as a source for plasma metabolome changes [17,18,19]. Oral antibiotics were administered via gavage (p.o.); parenteral administration was conducted via subcutaneous (s.c.) or intraperitoneal (i.p.) injection. The resulting metabolite profiles were compared to the profiles of the orally bioavailable antibiotic (roxithromycin), administered orally and parenterally [18,19,20]. Roxithromycin was shown to be absorbed from the blood into the intestinal lumen after parenteral administration [20]. Therefore, intestinal roxithromycin concentrations were assumed to be similar after oral and parenteral administration. All three antibiotics are broad-spectrum antibiotics with different activity spectra. The effects of oral antibiotic administration on the gut microbiome’s composition were characterized with a 16s rRNA community analysis. Comparing the plasma metabolite profiles of orally and parenterally antibiotic-treated rats provides a novel opportunity to identify systemic antibiotic-induced effects and distinguish these from microbiome-derived effects.

## 2. Results

### 2.1. Clinical Signs

Animals orally treated with roxithromycin showed slight salivation immediately after administration. Nothing abnormal was detected in the other treatment and control groups. As these clinical signs were not accompanied by a significant decrease in body weight relative to the feeding control, these clinical signs were interpreted as borderline effects and not indicative of systemic organ toxicity. Food consumption was measured for the entire cage (5 animals) and not for individual rats; hence, no statistical methods could be applied to evaluate statistical differences in food consumption. Relative changes in body weight and food consumption noted upon administration of the test compounds are shown in Table 1.

### 2.2. 16S rRNA Community Analysis

**Figure 1:**
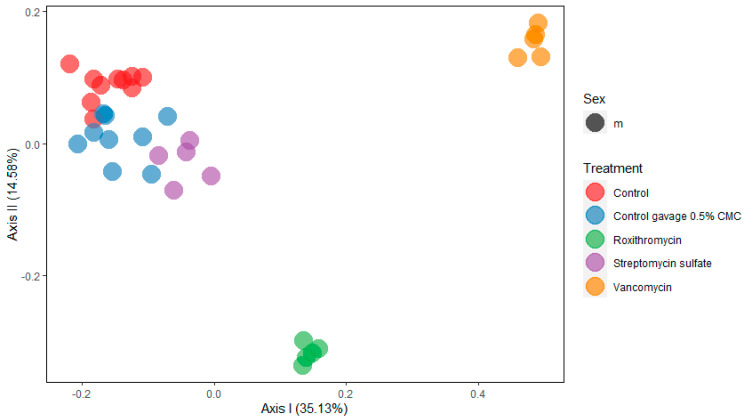
Principal coordinate analysis (PCoA) of the gut microbial community showing the treatment-related effect of oral administration of roxithromycin, streptomycin and vancomycin versus the feeding control and the vehicle control (0.5% carboxymethylcellulose (CMC) in drinking water).

To characterize the changes in the gut microbiome’s composition upon oral antibiotic administration, 16S rRNA gene sequencing was performed from the isolated bacterial DNA in the feces after 28 days of daily antibiotic administration. Figure 1 shows a principal coordinate analysis (PCoA) of gut microbial composition in orally antibiotic-treated and control rats. Roxithromycin and vancomycin show a clear and different treatment-related effect on the gut microbiome’s composition. Streptomycin shows only a weak separation from the controls.

### 2.3. Comparison of Plasma Metabolite Patterns after Oral and Parenteral Antibiotic-Administration

**Figure 2:**
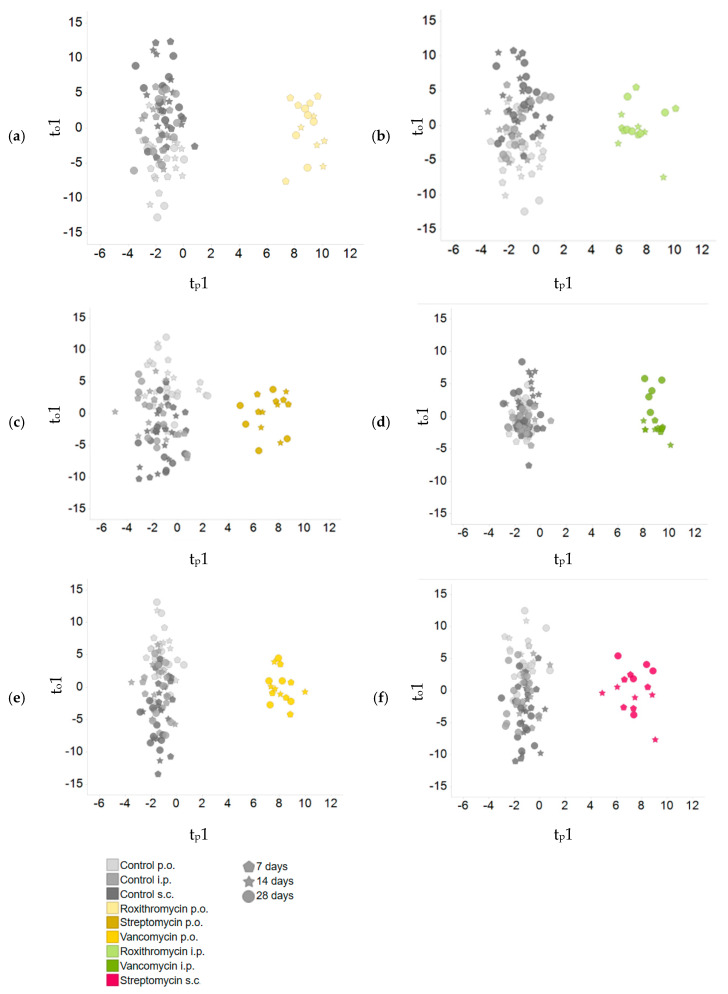
O-PLS-DA score plots. Individual models were generated by comparing samples from each antibiotic treatment and application route to all controls. (**a**) roxithromycin p.o. (**b**) roxithromycin i.p. (**c**) streptomycin p.o. (**d**) vancomycin i.p. (**e**) vancomycin p.o. (**f**) streptomycin i.p. Rats were either orally or parenterally dosed with vancomycin, streptomycin and roxithromycin (*n* = 5 per group). The control group received no treatment, the gavage control group received 0.5% CMC in drinking water and the intraperitoneal and subcutaneous control group were injected with a saline solution (*n* = 10 per group). Each dot represents a single plasma sample. The different days are represented by different point shapes. t_p_1, scores for first predictive component; t_o_1, scores for first orthogonal component; p.o., per os (by mouth); i.p., intraperitoneal; s.c., subcutaneous.

A principal component analysis (PCA) was conducted to visualize treatment effects on the plasma metabolome. No clear clusters were observed (see Supplementary Figure S1). To check whether the samples of a given treatment share a common metabolic difference from all the controls, a supervised orthogonal projection to latent structures discriminant analysis (O-PLS-DA) was performed. To characterize metabolic variation in the plasma metabolome, individual O-PLS-DA models were calculated to compare each antibiotic and administration route to all controls. Data from all time points were used to increase model power as there was no obvious clustering by time (see Supplementary Figure S1). For all six models, a good discrimination was observed between the antibiotic treatments and controls based on the first predictive and first orthogonal component; see Figure 2. The top analytes (here defined by having an absolute loading value of >0.125) from each model are highlighted in the corresponding loading plots (See Supplementary Figure S2). The focus is on these 55 top metabolites in further data analysis. A list with the ANOVA contrast t-values of all measured metabolites can be found in Supplementary Table S1.

**Figure 3:**
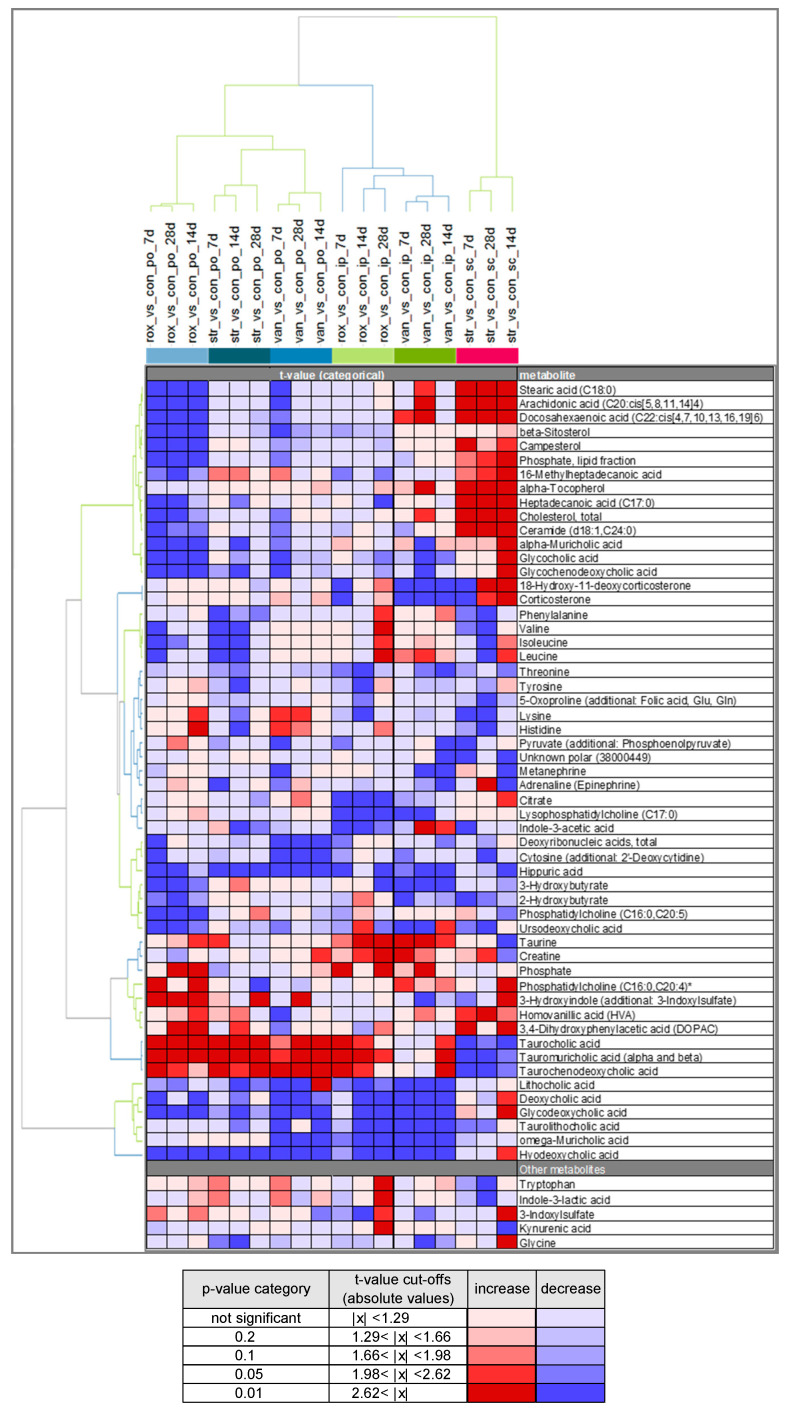
Hierarchical clustering analysis (HCA) of plasma metabolic profiles of antibiotic-treated male Crl/Wi(Han) rats compared to vehicle control animals. Animals were orally or parenterally dosed with roxithromycin, streptomycin or vancomycin for 28 days daily. Plasma samples were drawn on days 7, 14 and 28 (indicated by 7d, 14d, 28d). Coloring is based on the t-values. Input values are normalized against the respective vehicle control, and depicted in the axis label as antibiotic_vs_vehicle control_study day. rox, roxithromycin; str, streptomycin; van, vancomycin; con_po, per os vehicle control; con_ip, intraperitoneal vehicle control; con_sc, subcutaneous vehicle control. * additional: phosphatidylcholine (C18:2,C18:2).

Subsequently, a hierarchical clustering analysis (HCA) was performed to check for similarity between groups. The HCA (Figure 3) was based on ANOVA contrast t-values from the comparison of the individual antibiotic treatments versus the corresponding application control using the compiled list of 55 metabolites identified in the O-PLS-DA approach. The oral antibiotic treatments cluster together as expected. The s.c. streptomycin treatment is separated from the other parenteral antibiotic treatments. The difference in this treatment is most obvious for several lipid-related metabolites (increased) and the primary bile acids taurocholic acid, α- and β-tauromuricholic acid and taurochenodeoxycholic acid (all decreased). Interestingly, the i.p. roxithromycin and i.p. vancomycin treatments cluster more closely with the orally applied antibiotics than with s.c. streptomycin. This is most evident in the secondary bile acids (tauro)lithocholic acid, (glyco)deoxycholic acid, ω-muricholic acid and hyodeoxycholic acid (all decreased), and may indicate an effect of i.p. roxithromycin and i.p. vancomycin on the microbiome despite their parenteral administration.

All the treatments showed lower plasma hippuric acid (HA) concentrations in the antibiotic-treated animals relative to the respective vehicle control at multiple time points. A consistent yet weaker effect was the lower levels of threonine found for all antibiotic treatments. The indole-derivatives indole-3-acetic acid (IAA) and 3-hydroxyindole (with additional 3-indoxylsulfate—“additional” indicates that an analyte signal may be impacted by a different metabolite than the target metabolite) were identified as strong drivers of the separation, especially in the parenteral and oral roxithromycin treatment groups, respectively. Parenteral roxithromycin administration led to a downregulation of IAA, whereas oral roxithromycin induced an upregulation of 3-hydroxyindole (with additional 3-indoxylsulfate). Moreover, the list of prioritized metabolites contains phenylalanine, tyrosine and 2- and 3-hydroxybutyrate, all of them being predominantly downregulated after the antibiotic-treatments. The other measured indole-derivatives (indole-3-lactic acid, 3-indoxylsulfate and kynurenic acid) as well as glycine did not induce a strong separation between the antibiotic treatments and the controls and are shown under “Other metabolites”. Glycine is displayed because it is necessary for the formation of HA and is identified as a biomarker for nephrotoxicity. In the majority of groups glycine levels were slightly reduced. The list of prioritized metabolites contains one unconjugated primary bile acid, alpha-muricholic acid (α-MCA). The plasma concentrations of α-MCA were typically downregulated after antibiotic treatment, but s.c. streptomycin treatment induced an increase in α-MCA plasma levels. A similar pattern was observed for the glycine-conjugated primary bile acids glycocholic acid and glycochenodeoxycholic acid. The prioritized primary taurine-conjugated bile acids, taurocholic acid, α+β-tauromuricholic acid and taurochenodeoxycholic acid, were significantly upregulated after the oral administration of all three antibiotics and parenteral roxithromycin treatment. The secondary bile acids ursodeoxycholic acid, lithocholic acid, deoxycholic acid, glycodeoxycholic acid, taurolithocholic acid, ω-muricholic acid and hyodeoxycholic acid were predominantly downregulated after antibiotic treatment. Oral and parenteral vancomycin administration and parenteral roxithromycin administration induced the highest number of significant downregulations of secondary bile acids.

### 2.4. Variability in Plasma Bile Acid Levels

**Figure 4:**
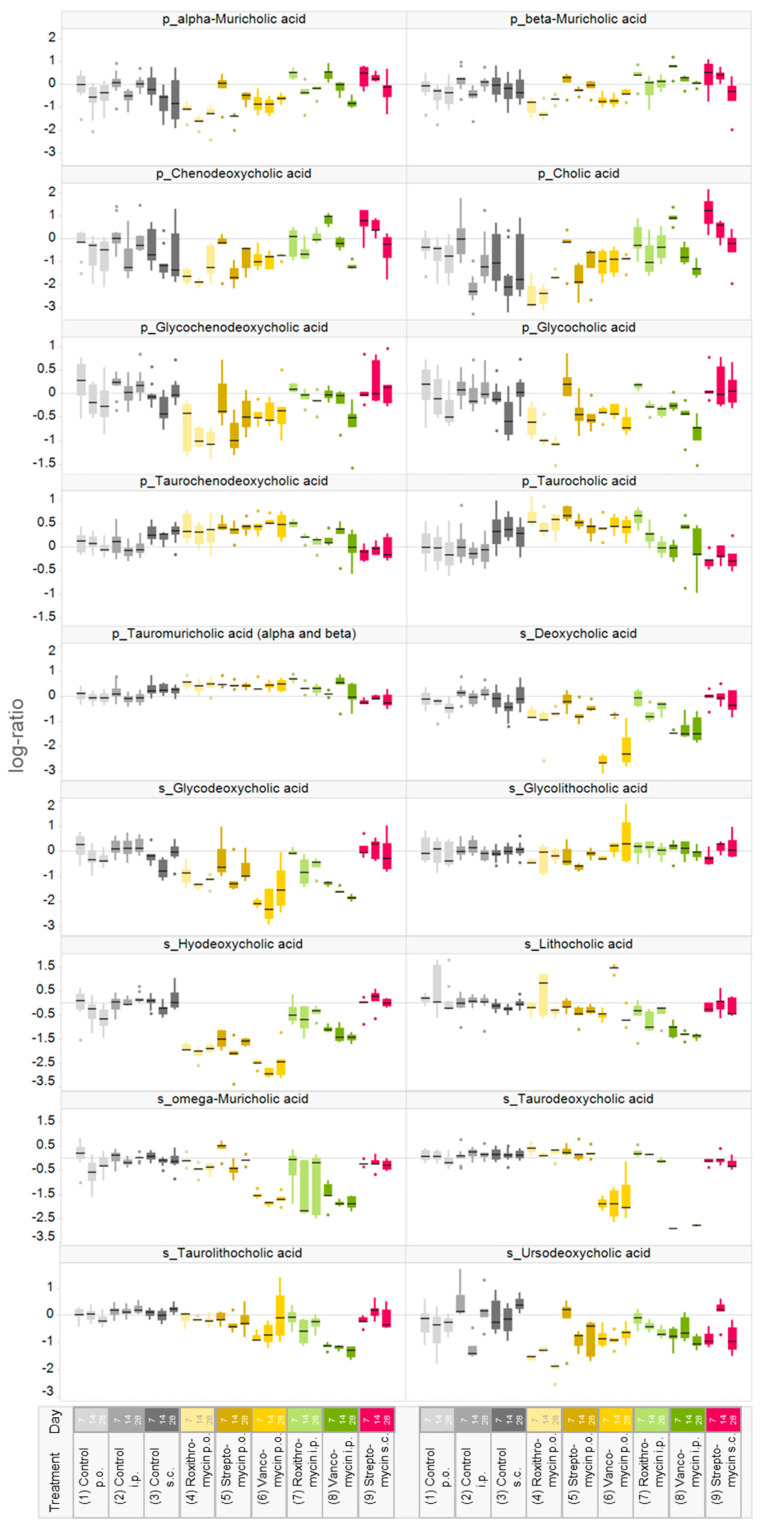
Plasma bile acid levels of male Crl/Wi(Han) rats treated orally or parenterally with vancomycin, streptomycin or roxithromycin. Input values are log10-transformed fold-changes relative to the feeding control. p_ indicates primary bile acid s_ secondary bile acid. The black horizontal line in each boxplot represents the group median value. p.o., per os (by mouth); i.p., intraperitoneal; s.c., subcutaneous.

It is expected that antibiotic treatments will affect bile acid levels. Based on the O-PLS-DA, a number of plasma bile acid levels can be identified as strong drivers of the separation between control and antibiotic-treated animals. For a more detailed evaluation, the distribution of all measured plasma bile acid levels was compared among the different treatments (Figure 4). Typically, antibiotic administration reduced variability in the plasma concentrations of primary bile acids, except for subcutaneous streptomycin. Oral and parenteral administration of roxithromycin and vancomycin, but not streptomycin, resulted in a decrease in the levels of primary unconjugated and primary glycine-conjugated bile acids. Decreases were typically stronger after oral than after parenteral treatment. The plasma levels of taurine conjugated primary bile acids (taurochenodeoxycholic acid, taurocholic acid and α+β-tauromuricholic acid) increased after oral treatment with all three antibiotics and parenteral roxithromycin treatment. The variability in plasma levels in secondary bile acids was smaller in vehicle control rats than in antibiotic-treated rats. The variability in secondary bile acids was typically smaller than in primary bile acids. Overall, the levels of several secondary bile acids were reduced by treatment with all three antibiotics, yet there were differences in the strength of the response among the antibiotics.

**Figure 5:**
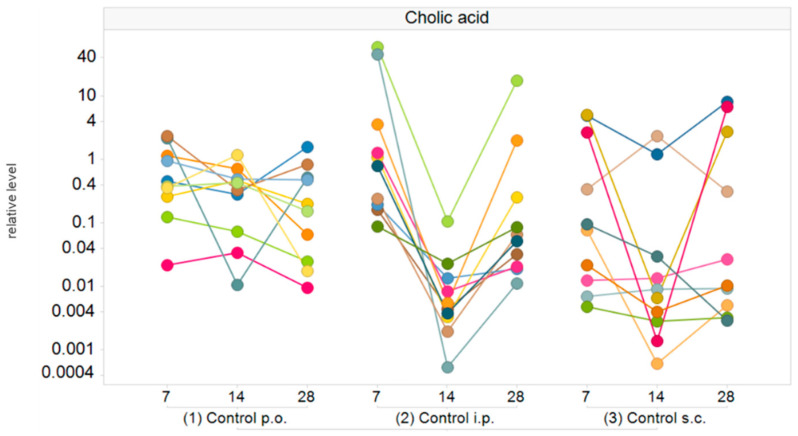
Relative cholic acid levels (normalized to the median value of the feeding controls) in plasma of male Crl/Wi(Han) rats in the different control groups (*n* = 10 per group) on days 7, 14 and 28. Values from the same animals are connected. p.o., per os (by mouth); i.p., intraperitoneal; s.c., subcutaneous.

In all treatment and control groups, inter- and intraindividual variabilities were observed in plasma bile acid levels. The variability of bile acid concentrations was most pronounced for cholic acid and taurocholic acid. Figure 5 exemplifies the relative cholic acid levels on days 7, 14 and 28 of the gavage and vehicle control animals. The values obtained from the same animals are connected. The variability between animals within the same group and day was up to a few thousand-fold, with the largest differences seen for control s.c. animals.

## 3. Discussion

Upon oral and parenteral treatment of male Crl/Wi(Han) rats over 28 days with antibiotics possessing different activity spectra and bioavailabilities, their plasma samples showed significant changes in metabolite levels compared to the control animals. As vancomycin and streptomycin are not or only poorly absorbed from the gut upon oral administration, it was assumed that systemic organ toxicity could be excluded and that the observed changes in the plasma metabolome were microbiome-derived. Further, after parenteral administration, these two antibiotics were expected not to enter the gastro-intestinal (GI) tract, and therefore not to directly affect the functionality of the gut microbiome. In contrast, roxithromycin is known to be readily absorbed from the gut and to enter the gut after parenteral administration, and was therefore used as a compound that might induce a mixed effect on the plasma metabolome.

We used a supervised approach (O-PLS-DA) to identify metabolites with a large discriminatory power between antibiotic treatment and controls (Figure 2). Subsequently, the similarities between groups were visualized using HCA (Figure 3). The HCA revealed a large similarity between the oral treatments. The intraperitoneally administered antibiotics clustered more closely with the orally administered antibiotics than with s.c. streptomycin. It was expected that intraperitoneal roxithromycin administration would lead to microbiome-derived effects on the metabolome, as this antibiotic is reported to be excreted from the blood stream into the gut [20]. In line with its non-bioavailability, no excretion was expected for parenterally administered vancomycin. The observed clustering of i.p. vancomycin with the oral treatments and i.p. roxithromycin, however, demonstrates that vancomycin induced some microbiome-derived effects upon i.p. administration. It is hypothesized that part of the intraperitoneal dose was taken up from the body cavity into the blood stream, transported to the liver and delivered to the intestinal lumen via biliary excretion [21]. Further research could elucidate whether the observed similarities between i.p. and oral antibiotic administration are caused by pharmacokinetic properties or by the administration route per se. The current results denote that only the effects of s.c. streptomycin are exclusively systemic.

The 16S rRNA community analysis shows that orally administrated roxithromycin and vancomycin induce different treatment-related effects, while no clear treatment-related effect of streptomycin was observed. Streptomycin is active only against aerobic bacteria, which constitute only a minor part of the gut microbiome [22]. The depletion of aerobic bacteria, however, can modify the gut microbiome’s composition of anaerobes, such as an increased abundancy of *Bacteroidacaea* and *Ruminococcaceae* [23]. The differences in the gut microbiome’s composition after oral roxithromycin and vancomycin treatment as observed in the current study confirm that these antibiotics have different activity spectra. The different activity spectra of antibiotics provide the opportunity to target undesired species in the gut microbiome. Pre- and probiotics have been proposed as solutions to restore disruptions in the bacterial population. Probiotics can introduce microbial strains that are beneficial for human health. Prebiotics can amend the proliferation of beneficial microbial strains. Combinations of antibiotics, probiotics and prebiotics could possibly provide a balanced approach for the prevention or treatment of various diseases, such as antibiotic-associated diarrhea and colitis, inflammatory bowel disease or acute gastroenteritis, while limiting or reversing the reduction in the number of beneficial microbes [24,25].

The shikimate pathway was discovered to be the biosynthetic pathway present in plants and microorganisms that forms the aromatic acids phenylalanine, tyrosine and tryptophan via their precursor chorismate [26]. Mammals were considered to lack this pathway, but recent studies have indicated the formation of metabolites derived from the shikimate pathway by the gut microbiome [27]. The products of the shikimate pathway can give rise to indoles, which fulfill many functions in microbial communities, such as quorum sensing, intercellular communication and signaling pathways [28]. They also play an important role in human health, which will be discussed later in this section. The combination of O-PLS-DA, ANOVA and HCA in the current study identified the downregulations of plasma tyrosine and phenylalanine, which are products of the shikimate pathway, as discriminants between oral streptomycin treatment and the controls. The third product of the shikimate pathway, tryptophan, was upregulated in the current study, although not significantly. A subgroup of enteric bacteria, mainly *Bacteroides* and *Alistipes,* expresses tryptophanase activity [29]. Hereby, tryptophan is converted into pyruvate, indole and ammonia. Tryptophanase activity in conventional mice can be elevated nearly twofold by dosing with tryptophan [30]. Germ-free mice show an increase in tryptophan levels [14], which indicates a loss of bacterial tryptophanase activity. It is hypothesized that tryptophanase activity is drastically downregulated in the current study, hence, no net result on tryptophan levels can be observed. Furthermore, pyruvate levels are not increased but rather decreased in most cases. The observed decreases in plasma tyrosine and phenylalanine after oral streptomycin treatment make it plausible that this antibiotic impairs the shikimate pathway.

Hippuric acid (HA) was downregulated after the administration of all three antibiotics via all administration routes (Figure 3). HA is a well-known urinary metabolite of host-microbial origin. Its precursor benzoate is derived from the microbial degradation of polyphenols from the diet or synthesized via the shikimate pathway [27,31]. Chorismate can be converted directly into benzoate or with phenylalanine as an intermediate [26]. Benzoate is subsequently conjugated with glycine in the kidney, liver and intestine to form HA [31,32,33]. Abnormal concentrations of HA in bodily fluids are associated with a variety of disease states. For example, morbidly obese and insulin-resistant patients show a remarkably lower urinary level of HA compared to healthy and lean individuals [34]. Decreased HA excretion is also associated with high blood pressure and atherosclerosis in rat models [31]. Antibiotic treatment is known to suppress the urinary excretion of HA in rats [35,36]. In germ-free mice, lower plasma levels of HA were observed compared to conventional animals [14]. The strongest HA decrease (up to around fivefold) in the current study was observed after oral antibiotic treatment. Oral antibiotic administration probably resulted in a reduced formation of benzoate and subsequent HA levels. Moreover, vancomycin and streptomycin can be nephrotoxic [37,38], hence, the HA decrease after parenteral administration could (partially) result from an impairment of the conjugation of benzoate in the kidney. Ryu and Kim [39] identified an increase in 3-hydroxybutyrate, citrate, creatine, glycine and lactate as sensitive plasma biomarkers for nephrotoxicity in Sprague Dawley rats. In the current study, plasma creatine levels were upregulated, while 3-hydroxybutyrate and citrate were predominantly downregulated after the antibiotic treatments. Lactate and glycine levels were not prioritized in the O-PLS-DA, yet sporadic significant upregulations in lactate (data not shown) and glycine were observed, but not at multiple time points. As no clinical signs and no consistent increases in plasma nephrotoxicity biomarkers were observed, it is assumed that the doses of vancomycin and streptomycin as administered in the current studies are not nephrotoxic. The authors hypothesize that a small amount of the parenterally administered dose ends up in the intestinal tract via biliary secretion and modulate the microbiome [21,40,41]. Thereby, the observed decrease in HA after both oral and parenteral administration indicates that HA is a sensitive indicator of gut microbial modulation.

Indole-3-acetic acid (IAA), a protein-bound uremic solute, was significantly downregulated at multiple time points after parenteral roxithromycin and oral streptomycin treatment. IAA is derived either from bacterial indole production or from dietary tryptophan degradation by both endogenous and bacterial cells. *Clostridium sporogenes*, *Clostridium bartlettii* and *Escherichia coli* are able to degrade tryptophan into IAA [42]. High IAA plasma levels were previously shown to induce the pro-inflammatory enzyme COX-2 and oxidative stress via the aryl hydrocarbon receptor (AhR) pathway in the endothelial cells of patients suffering from chronic kidney disease (CKD) and therefore are used for the prediction of mortality and cardiovascular events [43]. From the other indole-derivatives measured in the current study, 3-hydroxyindole (with additional 3-indoxylsulfate) is prioritized and significantly upregulated after oral roxithromycin treatment at multiple time points. In a previous study, 3-indoxylsulfate (IS) was identified only in the serum of conventional and not germ-free mice [14], hence, can be considered to be microbiome-derived. IS is a nephrotoxin that is formed in the liver from indole. IS accumulates in the blood of patients suffering from CKD [44]. Previously, it was hypothesized that treatment with vancomycin and streptomycin decreased the microbiome’s tryptophanase activity. In this way, the plasma concentrations of indole and indole metabolites would be reduced. However, in our study, after oral antibiotic treatment, not many significant downregulations in indole-derivatives were noticed. Our results indicate that IAA is the most sensitive indole-derivatized indicator of gut microbial modulation, as it was clearly downregulated after parenteral roxithromycin treatment.

The most abundant ontology class among the 55 metabolites prioritized in the O-PLS-DA is that of bile acids. These findings confirm that bile acids are strongly modulated by antibiotic administration. While bile acids were initially recognized as important for the emulsification of hydrophobic compounds, recently the role of bile acids as signaling molecules in glucose, lipid, energy and bile acid metabolism has become clearer. Bile acids activate FXR to induce fibroblast growth factor (FGF) 15, which ultimately leads to inhibition of the transcription of CYP7A1 and CYP8B1, which facilitate the formation of primary bile acids from cholesterol in the liver [8]. The current results demonstrate that oral roxithromycin and vancomycin treatment decreased the levels of primary bile acids, while the taurine conjugates of primary bile acids were increased with oral administration of all three antibiotics and parenteral roxithromycin administration. It is shown in the literature that taurine-conjugated bile acids dominate the bile acid profiles in the liver, kidneys, heart and plasma in germ-free rats, while unconjugated bile acids dominate these bile acid profiles in conventional rats [45]. Since in the current study the increase in taurine conjugates is nearly exclusively observed for antibiotics that enter the gut, this is considered a robust indicator of microbiome modulation. The liver secretes taurine or glycine conjugates of the bile acids into the bile canaliculus. In the intestine, these conjugated bile acids are deconjugated to primary bile acids by bile salt hydrolases (BSH), which are expressed by *Bacteroides*, *Clostridium*, *Lactobacillus*, *Bifidobacterium* and *Listeria* species. Antibiotic-induced disturbance of this deconjugation pathway would lead to the accumulation of conjugated bile acids and a decrease in primary bile acids. Secondary bile acids are formed from primary bile acids by a wide range of microbial reactions [46]. Hence, reduced levels of primary bile acids would lead to reduced levels of secondary bile acids, which is confirmed by the current study. As the decreases in secondary bile acids were typically stronger after oral than after parenteral treatment, they seem very sensitive to alterations in the microbiome. Studies have shown that tauromuricholic acid is a potent antagonist that inhibits FXR induction of FGF15 in the ileum [47]. Thereby, not only does the lack of microbial deconjugation by BSH lead to the increased levels of primary bile acids, but also the increased levels of tauromuricholic acid lead to increased primary bile acid synthesis via the inhibition of FXR.

Profound variability in the bile acid levels both between and within animals was observed (Figure 4 and Figure 5). The technical variability (i.e., variability introduced by the analytical measurement) in the current study was determined to be about 10% and therefore much lower than the observed biological variability. Biological variability in bile acid levels is a well-known phenomenon. Bile acids are known to undergo cyclic daily variation in both rodents [48,49] and humans [50]. Cyclic daily variation is strongly influenced by the circadian rhythm, an endogenously generated rhythm with a period close to 24 h which is tightly coupled to the light-dark cycle and food intake [51]. Possibly, the overnight fasting in the current experiment disrupted the food intake and as a consequence the bile acid metabolism, which might explain part of the observed variability between and within animals. Bile acid biosynthesis predominantly takes place in hepatic cells via the multistep oxidation of cholesterol. Prior to secretion into the bile canaliculus, bile acids are conjugated by addition of taurine or glycine. After secretion in the intestinal lumen, bile salts are modified by gut bacteria and recycled via enterohepatic circulation. Therefore, any change in the production of primary bile acids will affect their intestinal concentration, which in turn affects the production of secondary bile acids. As an altered bile acid pool is implicated in various disease states [12,13], the current study demonstrates the need to better understand the influence of environmental factors on the gut microbiome–liver axis.

## 4. Materials and Methods

Animal handling, treatment and clinical examinations were performed according to the Organisation for Economic Co-operation and Development (OECD) guideline 407. Briefly, Wistar rats (CrI/WI(Han)) were supplied by Charles River, Germany, and were approximately 70 days old at the beginning of the studies. The animals were kept in enriched cages at a temperature of 20 to 24 °C, a relative humidity of 30% to 70%, 15 air changes per hour and a 12 h light/12 h dark cycle. Ground Kliba mouse/rat maintenance diet was supplied by Provimi Kliba SA, Kaiseraugst, Switzerland. The diet and drinking water were available ad libitum and regularly assayed for chemical contaminants and the presence of microorganisms. Food and water intake were restricted before blood sampling. Metabolite profiling of plasma samples was performed for all control and treated animals. On study day 28, feces were sampled from the animals that received oral antibiotic treatment plus the corresponding controls, and the gut microbial community was analyzed.

### 4.1. Treatment of the Animals

The antibiotics were administered daily for 28 days to treatment groups consisting of five male animals. Dose levels, routes of administration and the form of preparation of the antibiotics are summarized in Table 2. A feeding control group was included in each study (10 males). This manuscript combines data from multiple previously performed unpublished studies, hence, two different parenteral administration routes were used, i.e., intraperitoneal (i.p.) and subcutaneous (s.c.). Besides feeding controls, vehicle controls for i.p., s.c. and gavage administration were tested (*n* = 5 per group) to examine the effect of the vehicle and the administration procedure on the plasma metabolome. The feeding controls served as a common reference present in all studies and were used to normalize between studies and the different vehicle controls and treatments (this is required for the semi-quantitative metabolome analysis to account for general study-to-study differences, data not shown). The control groups are summarized in Table 3. The i.p. and s.c. vehicle control groups were injected daily with a saline solution. The control animals in the gavage group received 0.5% carboxymethylcellulose (CMC) Tylose CB30000 in drinking water via gavage. The treated and vehicle control animals were normalized to feeding controls according to time point and study and compared. In former studies, the metabolome of control groups with different administration routes had been compared. Rats gavaged with 0.5% CMC had less than 5% significantly altered metabolites compared to diet controls (Welch *t* test, *p* < 0.05). This is regarded as an incidental change, which did not interfere with antibiotic-related effects on the treatment groups in our study.

### 4.2. Ethics Statement

The studies were approved by the BASF animal welfare body, which received permission from the local authority, the Landesuntersuchungsamt Rheinland-Pfalz (reference number 23177-07). The laboratory has been AAALAC (Association for Assessment and Accreditation of Laboratory Animal Care International)-certified since 2007.

### 4.3. Blood Sampling

On study days 7, 14 and 28, after a fasting period of 16–20 h and 1 day after the last administration of the test substances, blood samples were taken from the retro-orbital sinus in all rats under isoflurane anesthesia. 1.0 mL of blood was immediately transferred with a 1 mL pipette tip into an Eppendorf tube containing 10 µL of 10% Titriplex III (K-EDTA) solution. The blood samples were centrifuged (10 °C, 2000× *g*, 10 min) and the K-EDTA plasma was separated. The K-EDTA plasma samples were covered with nitrogen and frozen at −80 °C until the performance of metabolite profiling. Day 0 analysis were not performed, because the frequent sampling of blood (days 0, 7, 14 and 28) was considered to be too high a stress factor for the animals and could have impacted their state of health and hence the validity of the study. As the animals used were still in their growth phase, the amount of blood at day 0 was less than on the following days. The concurrent control animals were considered to be sufficiently robust.

### 4.4. Clinical Examinations

All animals were checked daily for clinically abnormal signs and mortalities. Food consumption and body weight were determined on study days 6, 13 and 27, with an additional measurement for body weight on day 0. Body weight was determined before the start of the test period for the randomization of the animals. At the end of the treatment period, the animals were sacrificed by decapitation under isoflurane anesthesia. Metabolome evaluation of the blood samples was performed for all control and treated animals. Feces samples were carefully removed from the rectum during necropsy on day 28 after a fasting period of 16–20 h and one day after the last administration of the test substances. The samples were collected in pre-cooled (dry-ice) vials, immediately snap-frozen in liquid nitrogen and stored at −80 °C until the community analysis was performed.

### 4.5. Metabolite Profiling

#### Broad Profiling

Mass-spectrometry-based broad metabolite profiling of the K-EDTA plasma samples was performed by GC-MS (gas chromatography-mass spectrometry) and LC-MS/MS (liquid chromatography-tandem mass spectrometry) techniques as described in detail in previous work [52,53,54,55] and below.

Online SPE–LC–MS/MS (solid phase extraction–LC–MS/MS; SPARK Holland Symbiosis) was applied for the determination of catecholamine and steroid hormone levels. Proteins were removed from 60 µL K-EDTA plasma samples by precipitation using 200 µL acetonitrile. Subsequently, polar and non-polar fractions were separated for both GC-MS and LC-MS/MS analysis by adding water and a mixture of ethanol and dichloromethane (1:2, *v*:*v*). For the GC-MS analysis (CTC GC PAL, Agilent 6890 GC gas chromatograph, 5973 MSD mass spectrometer), the non-polar fraction was treated with methanol under acidic conditions to yield the fatty acid methyl esters derived from both free fatty acids and hydrolyzed complex lipids. The non-polar and polar fractions were further derivatized with O-methyl-hydroxylamine hydrochloride and pyridine to convert oxo-groups to O-methyl-oximes, and subsequently with a silylating agent, before analysis [56]. For the LC-MS analysis (Agilent 1100, AB Sciex 4000), both fractions were reconstituted in appropriate solvent mixtures. High-performance liquid chromatography (HPLC) was performed by gradient elution on reversed-phased separation columns. Mass spectrometric detection technology was applied, allowing targeted and high-sensitivity MRM (Multiple Reaction Monitoring) profiling in parallel to a full-screen analysis as described in patent WO2003073464 [57]. For GC-MS the acquisition in scan mode *m*/*z* 15–600 for polar compounds and *m*/*z* 40–600 for lipid compounds was applied. For LC-MS, MRM and a Q3 scan of *m*/*z* 100–1000 were used. MRM was determined for all analytes using solutions of the authentic standard.

GC-MS conditions: CTC GC PAL, Agilent 6890 GC gas chromatograph, 5973 MSD mass spectrometer, gradient: 70 to 340 °C and carrier gas: helium. For polar compounds a J&W DB-XLB and for lipid compounds an Agilent HP-5MS were used. Both columns provide 30 m length, an inner diameter (ID) of 0.25 mm and 0.25 µm film thickness.

LC-MS conditions: an LC-MS system consisting of an Agilent 1100 HPLC system coupled to an AB Sciex API 4000 mass spectrometer equipped with an ESI ion source running in negative ionization mode at a source temperature of 600 °C and ion spray voltage of −4000 V for acquiring data after the injection of 10 µL polar compounds containing extract to be chromatographically separated on a C18 reversed-phased column (Grom-Sil 80 ODS7 PH, 4 µm, 60 × 2.0 mm, 5 °C) and a gradient elution profile (0.0 min: 100% A; 0.5 min: 100% A; 3.5 min: 0% A; 4.5 min: 0% A; 4.6 min, 100% A; 6.0 min: 100% A) running at 200 µL/min where solvent A consisted of deionized water with 0.1 M ammonium formate (99/1; *w*/*w*) and solvent B consisted of acetonitrile with 0.1 M ammonium formate (99/1; *w*/*w*). The LC-MS system for the analysis of lipid compounds consisted likewise of an Agilent 1100 HPLC system coupled to an AB Sciex API 4000 mass spectrometer equipped with an ESI ion source, but running in positive ionization mode at a source temperature of 400 °C and ion spray voltage of +5500 V for acquiring data after the injection of 5 µL of lipid compounds containing extract to be chromatographically separated on a C18 reversed-phased column (Thermo Betasil C18, 5 µm, 50 × 2.1 mm, 35 °C) and a gradient elution profile (0.0 min: 100% A; 0.5 min: 60% A; 5.5 min: 0% A; 6.0 min: 0% A; 6.1 min: 100% A; 7.0 min: 100% A) running at 200 µL/min where solvent A consisted of methanol, deionized water, 2-methoxy-2-methylpropan (MTBE) and formic acid (100/25/5/0.6; *w*:*w*:*w*:*w*) and solvent B consisted of MTBE, methanol, deionized water and formic acid (100/7.7/1.6/0.5; *w*:*w*:*w*:*w*).

For GC-MS and LC-MS/MS profiling, data were normalized to the median of reference samples which were derived from a pool formed from aliquots of all samples to account for inter- and intra-instrumental variation. In plasma, 274 semiquantitative metabolites could be analyzed using the single peak signal of the respective metabolite and a normalization strategy according to the patent WO2007012643A1 [58] resulting in ratio values representing the metabolite change in treated versus control animals. 248 plasma analytes were chemically identified and 26 were structurally unknown. This list of 274 metabolites contains metabolites measured more than once among the different analytical methods (e.g., LC- and GC-MS, lipid and polar phases). These metabolites were used to confirm the validity of the measurements. Six metabolites were removed due to many missing values. The duplicate-free list from broad profiling included 196 metabolites.

### 4.6. Targeted Bile Acid Analysis

The bile acid concentration of plasma samples obtained from orally treated rats was measured as described by Behr and Slopianka [59]. The bile acid analysis of samples obtained from parenterally treated, feeding and vehicle control rats was performed with ultra-high-performance liquid chromatography-electrospray ionization-MS/MS (UHPLC–ESI-MS/MS) consisting of a Spark Holland UHPLC system coupled with an SCIEX 5500 Triple Quad™ LC-MS/MS system equipped with an ESI ion source, which enabled the measurement of a total number of 22 bile acids. To ensure accuracy and precision, the method provided 7 calibration standards, a mixture of 9 isotope-labeled internal standards, and a quality control sample. A total of 10 µL of plasma was added together with 10 μL of internal standards mixture onto filter spots suspended in the wells of a 96-well filter plate (PALL AcroPrep, PTFE 0.2 µm) fixed on top of a deep-well plate and extracted with 100 μL methanol by shaking at 600 rpm for 20 min on an Eppendorf ThermoMixer C (Eppendorf AG, Hamburg). The elution of the methanol extracts was performed by centrifugation (5700 rpm, 5 min) into the lower receiving deep-well plate, which was then detached from the upper filter plate. After adding 60 μL Milli-Q^®^ water to the extracts and shaking briefly (600 rpm, 5 min), the 96-well plate containing the samples was analyzed by LC-MS/MS. All target isobaric bile acids were baseline-separated under UHPLC conditions based on a previously described method [60]. Briefly, UHPLC systems were used at a flow rate of 0.7–1 mL/min. Mobile phase A was water with 0.02% formic acid and 10 mM ammonium acetate and mobile phase B was 30% (*v*/*v*) acetonitrile/methanol with 0.02% formic acid and 10 mM ammonium acetate. The gradient program initially started at 35% B, increased to 100% B in 3.5 min, was held at 100% B for 0.5 min, decreased to 35% B and was held for 1.0 min, enabling a short runtime of 5 min. Chromatographic separation was performed with a proprietary reversed-phased UHPLC analytical column (Ascentis Express C18 2.7 µm 50 × 2.1 mm) kept at 50 °C. An injection volume of 5 μL was used. Mass spectrometric detection was accomplished with electrospray ionization in negative ion mode. Two MRM transitions were used for each target bile acid for quantitative evaluation; 22 bile acids were measured. Bile acids with more than 50% missing values were discarded from further analysis, resulting in a final list of 18 bile acids.

### 4.7. Community Analysis

On study day 28, feces were sampled from the animals which received oral antibiotic treatment. DNA was isolated from the fecal samples using an InnuPREP stool DNA Kit (Analytik Jena, Jena, Germany) according to the manufacturer’s instructions. Based on observations made during the process, the incubation temperature for the cells’ lysis was lowered to 75 °C. DNA yield and integrity were assessed using a Nanodrop. Samples were sent to IMGM^®^ laboratories (Martinsried, Germany) for polymerase chain reaction (PCR), library preparation and sequencing. DNA was amplified using 16S V3–V4 primers (Bakt_341F: 5′-CCTACGGGNGGCWGCAG-3′ and Bakt_805r: 5′-GACTACHVGGGTATCTAATCC-3′). Sequencing was performed on the IlluminaMiSeq^®^ next-generation sequencing system (Illumina Inc., San Diego, CA, USA). Signals were processed to FASTQ files and the resulting 2 × 250 bp reads were demultiplexed using the MiSeq^®^ Reporter software version 2.5.1.3.

### 4.8. Bioinformatics

Forward and reverse primers corresponding to the sequences 5′CCTACGGGNGGCWGCAG-3′ and 5′-GACTACHVGGGTATCTAATCC-3′ were trimmed from the raw reads using Cutadapt [61] and forward/reverse read pairs that did not contain both primers were removed. A table of amplicon sequence variants (ASV) was obtained by denoising using QIIME2’s (Quantitative Insights Into Microbial Ecology) “dada2 denoise-single” command [62,63]. ASVs that did not have a count of at least 1 in 2 or more samples were excluded from further analysis. Counts were normalized using the relative rank. A PCoA was computed based on the Bray distance matrix at the taxonomic family level. The plot was made using the RAM (R for Amplicon-Sequencing-Based Microbial-Ecology) package. More details can be found in the R markdown file in the Supplementary Materials.

### 4.9. Statistics

The data were analyzed by univariate and multivariate statistical methods. ANOVA was applied using the R package “nlme” with treatment (the combination of substance and application route) and day as fixed and the animal-identifier as random factor to compare metabolite levels between treatments with respective controls and days [64]. Hereby, ratios were calculated that are referred to as “relative abundance in plasma.” Whenever “significantly” is written, “statistically significantly” is meant. For the metabolite data, Principal Component Analysis (PCA) and O-PLS-DA analyses were performed using the commercial software Simca (version 15, Sartorius-Stedim Data Analytics AB, Umeå, Sweden). For multivariate analysis, log10-transformed, feeding control-normalized metabolite values were used as input. Metabolites and samples with >50% missing values were discarded. The average of all samples was centered, i.e., set to 0. Scaling to unit variance was applied, i.e., the standard deviation of all samples was set to 1. For O-PLS-DA, six separate models (3 antibiotics times 2 application routes) were calculated. In each case, all controls (irrespective of the application route) were defined as the first group and the treatment (antibiotic plus application route) as the second group. This was done to ensure that the model would differentiate the treatment from all the controls. In order to have a higher power, data from all three days were combined. Hierarchical clustering analysis (HCA) was done in TIBCO Spotfire (version 6, TIBCO, Palo Alto, CA, USA) using t-values derived from ANOVA as input with the following settings (for both row and column dendrograms): Ward’s clustering method with half-square Euclidean as distance measure, input average rank as ordering weight and Z-score normalization.

## 5. Conclusions

The downregulation of HA following both oral and parenteral administration of all three antibiotics indicate that HA is a sensitive indicator of microbiome modulation, as a small amount of the antibiotic is assumed to be secreted via the bile in the gut after parenteral administration. Furthermore, the bile acid pool is very sensitive to antibiotic administration. Besides antibiotic-induced depletion of microbial species responsible for the conversion of bile acids, potential feedback mechanisms regarding bile acid production might alter the plasma metabolome. Bile acid levels show large variability within and between animals. Reduction of this variability, e.g., by the development of standardized in vivo and in vitro models, would increase the statistical quality of the bile acid data. Revealing meaningful interactions between the microbiome and bile acid metabolism will offer new opportunities for better treatment and prevention of microbiome-associated diseases, as well as improved evaluations of the influence of environmental factors on this system.

## Figures and Tables

**Table 1:** Fold changes in body weight and food consumption of Crl/Wi(Han) rats (*n* = 5) dosed for 28 days compared to the corresponding feeding controls (*n* = 10 per group). None of the relative decreases in body weight compared to corresponding feeding controls were statistically significant (*p* < 0.05).

Antibiotic	Route of Administration	Day	Body Weight	Food Consumption
Vancomycin	Gavage	6	0.99	0.88
		13	0.97	0.93
		27	0.95	0.93
	Intraperitoneal	6	0.98	0.91
		13	0.99	1.08
		27	0.99	1.20
Streptomycin	Gavage	6	0.97	0.92
		13	0.96	0.92
		27	0.95	0.97
	Subcutaneous	6	1.00	0.97
		13	0.99	1.02
		27	1.00	1.13
Roxithromycin	Gavage	6	0.98	0.85
		13	0.96	0.86
		27	0.96	1.04
	Intraperitoneal	6	0.97	0.92
		13	0.96	0.99
		27	0.97	1.05

**Table 2:** Compounds used, class of antibiotics, routes of administration, dose levels and form of preparation.

Treatment	Class of Antibiotics	Route of Administration	Dose/Day (mg/kg Body Weight)	Form of Preparation
Vancomycin	Glycopeptide	Gavage	50	In ultra-pure water
		Intraperitoneal	50	In saline
Streptomycin	Aminoglycoside	Gavage	100	In drinking water containing 0.5% CMC
		Subcutaneous	120	In saline
Roxithromycin	Macrolide	Gavage	200	In drinking water containing 0.5% CMC
		Intraperitoneal	20	In saline

**Table 3:** Description of (vehicle) controls.

Controls	Vehicle
Feeding	None
Gavage	0.5% CMC in drinking water
Intraperitoneal	Saline solution
Subcutaneous	Saline solution

## Supplementary Materials

The following are available online at https://www.mdpi.com/2218-1989/10/6/242/s1, Figure S1: PCA of treatment effect of vancomycin, streptomycin and roxithromycin on the plasma metabolome of male rats, Figure S2: O-PLS-DA loading plots; Table S1: ANOVA results for the full metabolite list.

## Abbreviations

AAALAC	Association for Assessment and Accreditation of Laboratory Animal Care International
CKD	chronic kidney disease
CMC	carboxymethylcellulose
Da	Dalton
ESI	electrospray ionisation
FGF	fibroblast growth factor
FXR	farnesoid X receptor
GC	gas chromatography
GCDCA	glycochenodeoxycholic acid
HA	hippuric acid
HCA	hierarchical clustering analysis
i.p.	intraperitoneal
IAA	indole-3-acetic acid
IS	3-indoxylsulfate
ID	inner diameter
LC	liquid chromatography
MS	mass spectrometry
MRM	multiple reaction monitoring
MTBE	2-Methoxy-2-methylpropan
OECD	Organisation for Economic Co-operation and Development
O-PLS-DA	orthogonal projection of latent structures
p.o.	per os, by mouth
PCA	principal component analysis
PCoA	principal coordinate analysis
PCR	Polymerase chain reaction
s.c.	subcutaneous
SPE	solid phase extraction
TCA	taurocholic acid
TCDCA	taurochenodeoxycholic acid
TGR5	trans-membrane-bound G-protein-coupled receptor
UHPLC-ESI	ultra-high-performance liquid chromatography-electrospray ionization
VDR	vitamin D receptor
QIIME	Quantitative Insights Into Microbial Ecology
α-MCA	alpha-muricholic acid
