# Supplementary material for: Antibiotic-Induced Changes in Microbiome-Related Metabolites and Bile Acids in Rat Plasma
Source: Metabolites. 2020 Jun 11;10(6):242. doi: 10.3390/metabo10060242 (PMC7344402; doi:10.3390/metabo10060242)
Supplement: Supplementary file 1 [file metabolites-10-00242-s001.zip › CommAnalysis_Veronique.pdf]

Community Analysis

VG

2020 M03 06

Data Import and pre-processing

Imported libraries (primarily RAM and Vegan) and helper functions.

Data Read-in

The raw data was provided by Hunter, C. using dada2 processing pipeline on paired end. The data includes 16S positive and negative controls which were removed during the read-in process.

Hunter provided following information for the adaptation of the dada2 processing:

The changes between the two are as follows:

- Updated denoising workflow
- Reads merged instead of single end
- Different taxonomy assignment tool and database (old: sintax to SILVA LTP; new: RDP classifier to RDP database)
- These are both good assigners and databases the current denoising workflow is just using RDP. The results will be a bit different because of the tool changes and because we are classifying a longer fragment of sequence this time.

In general, the two datasets look comparable. I recommend we continue with the paired end dataset. The only downside to doing so is that the sequencing depth is less. However, I've ran a rarefaction analysis and we have plenty of depth to characterize the gut microbiome so that is a non-issue.

```
path <- '~/My Dataspace/Datasets/Microbiome/20190827/'
files <- c('sample_metadata.tsv', 'taxonomy.tsv', 'table.cleaned.tsv', 'tree.nwk')
micro_raw_data <- read_data(path, files, removeControls = T)
```

Read in 248 samples.

Data Cleanup

The following 3 animals were removed from analysis based on the downstream analysis:

- Vancomycin, Male, 27
- Streptomycin sulfate, Male, 36
- Clindamycinhydrochlorid, Female, 129 These samples clustered incorrectly and show strong differences in compositions as compared to other animals in the group.

```
micro_raw_data <- removeSpurious(micro_raw_data, F)
```

Number of samples: 245. Number of ASVs: 43463.

Subsetting to required set

Vancomycin, Streptomycin Sulfate and Roxithromycin

```
sid <- micro_raw_data$metadata[!(STUDY_NAME == 'MOA89' & SEX == 'm' & DOSE != 'HIGH'), `sample-id`]
micro_raw_data$freq[, (sid) := NULL]
micro_raw_data$metadata <- micro_raw_data$metadata[(STUDY_NAME == 'MOA89' & SEX == 'm' & DOSE != 'HIGH'

# Remove empty rows
micro_raw_data$freq <- micro_raw_data$freq[rowSums(micro_raw_data$freq[, -c('featureID')]) > 0]
micro_raw_data$taxonomy <- micro_raw_data$taxonomy[featureID %in% micro_raw_data$freq$featureID]

micro_raw_data$metadata$COMPOUND <- factor(micro_raw_data$metadata$COMPOUND)
micro_raw_data$metadata$COMPOUND <- relevel(micro_raw_data$metadata$COMPOUND, ref = 'Control')
```

Number of samples: 34. Number of ASVs: 6540.

Data Processing

Data Cleanup

As a first step, ASVs with low reads are removed.

```
a = 1
k = 2
```

The following clean-up are made: - k over a filter: Removed all ASVs that have do not have count 1 or more in atleast 2 samples.

```
data_filtered <- copy(micro_raw_data)
data_filtered <- filter_k_over_a(data_filtered, a, k, T)
```

```
##    Samples Features  Reads
## 1:      34     6540 437158
## 2:      34      855 398009
```

```
# RAM object of filtered data.
ram_filtered <- getOTU(data_filtered)
```

```
## Warning in valid.taxonomy(list(otu = otu)): Format of the taxonomy column is
## good
```

```
## [1] "34 samples exist in otus and metadata!"
```

Number of samples: 34. Number of ASVs: 855.

Analysis

PCoA

PCoA based on gower using filtered data.

Standardization using relative ranks - which is able to account for differences in library sizes.

The distances are computed based on Gower dissimilarity index.

```
pcoa.plot(ram_filtered$otu, is.OTU = T, ram_filtered$meta, factors = c(Sex = 'SEX', Treatment = 'COMPOU
          rank = 'f', stand.method = 'rrank', dist.method = 'gower', sample.labels = F, top = 0, bw = F
```

```
## Scale for 'shape' is already present. Adding another scale for 'shape', which
## will replace the existing scale.

## Scale for 'colour' is already present. Adding another scale for 'colour',
## which will replace the existing scale.
```

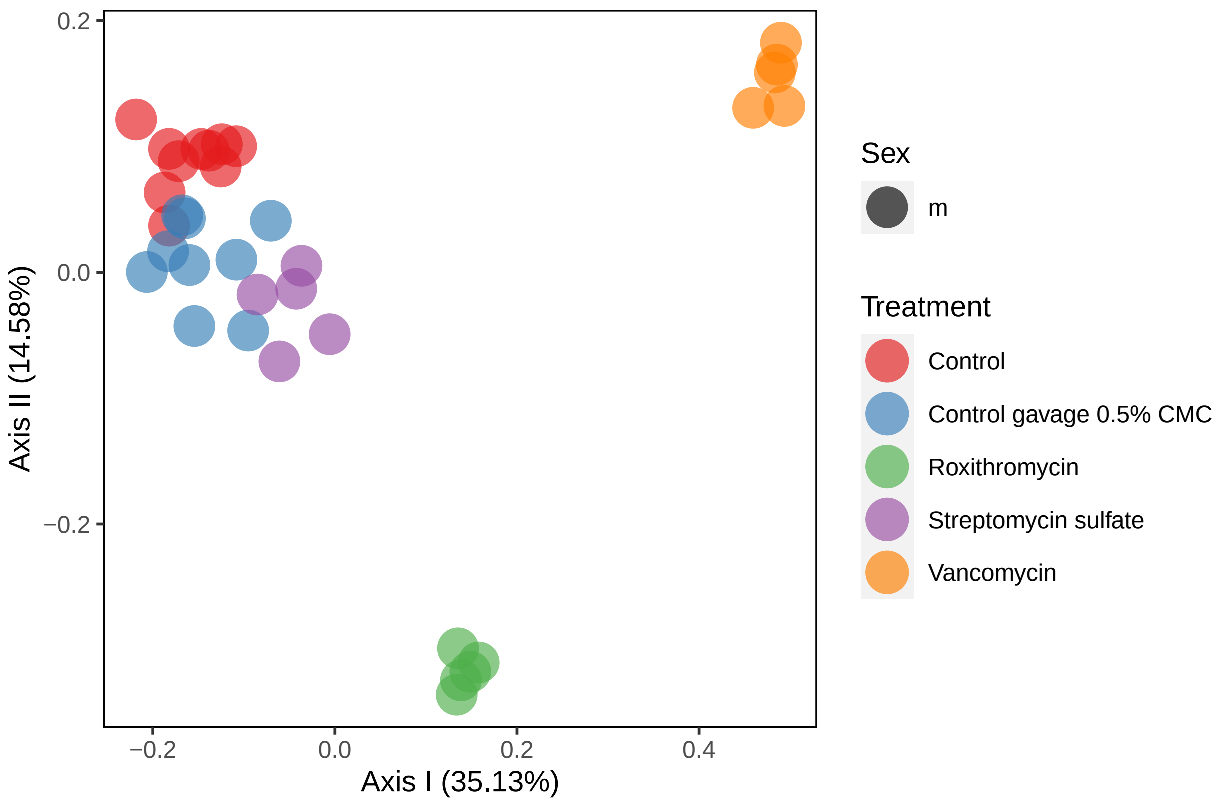

References

- Wen Chen, Joshua Simpson and C. Andre Levesque (2018). RAM: R for Amplicon-Sequencing-Based Microbial-Ecology. R package version 1.2.1.7. https://CRAN.R-project.org/package=RAM
- Jari Oksanen, F. Guillaume Blanchet, Michael Friendly, Roeland Kindt, Pierre Legendre, Dan McGlinn, Peter R. Minchin, R. B. O'Hara, Gavin L. Simpson, Peter Solymos, M. Henry H. Stevens, Eduard Szoecs and Helene Wagner (2019). vegan: Community Ecology Package. R package version 2.5-6. https://CRAN.R-project.org/package=vegan
