# Supplementary material for: Antibiotic-Induced Changes in Microbiome-Related Metabolites and Bile Acids in Rat Plasma
Source: Metabolites. 2020 Jun 11;10(6):242. doi: 10.3390/metabo10060242 (PMC7344402; doi:10.3390/metabo10060242)
Supplement: Supplementary file 1 [file metabolites-10-00242-s001.zip › Appendix A.docx]

Antibiotic-induced changes in microbiome-related metabolites and bile acids in rat plasma

Appendix A

| a) | PC1 (15%)  PC2 (7%) 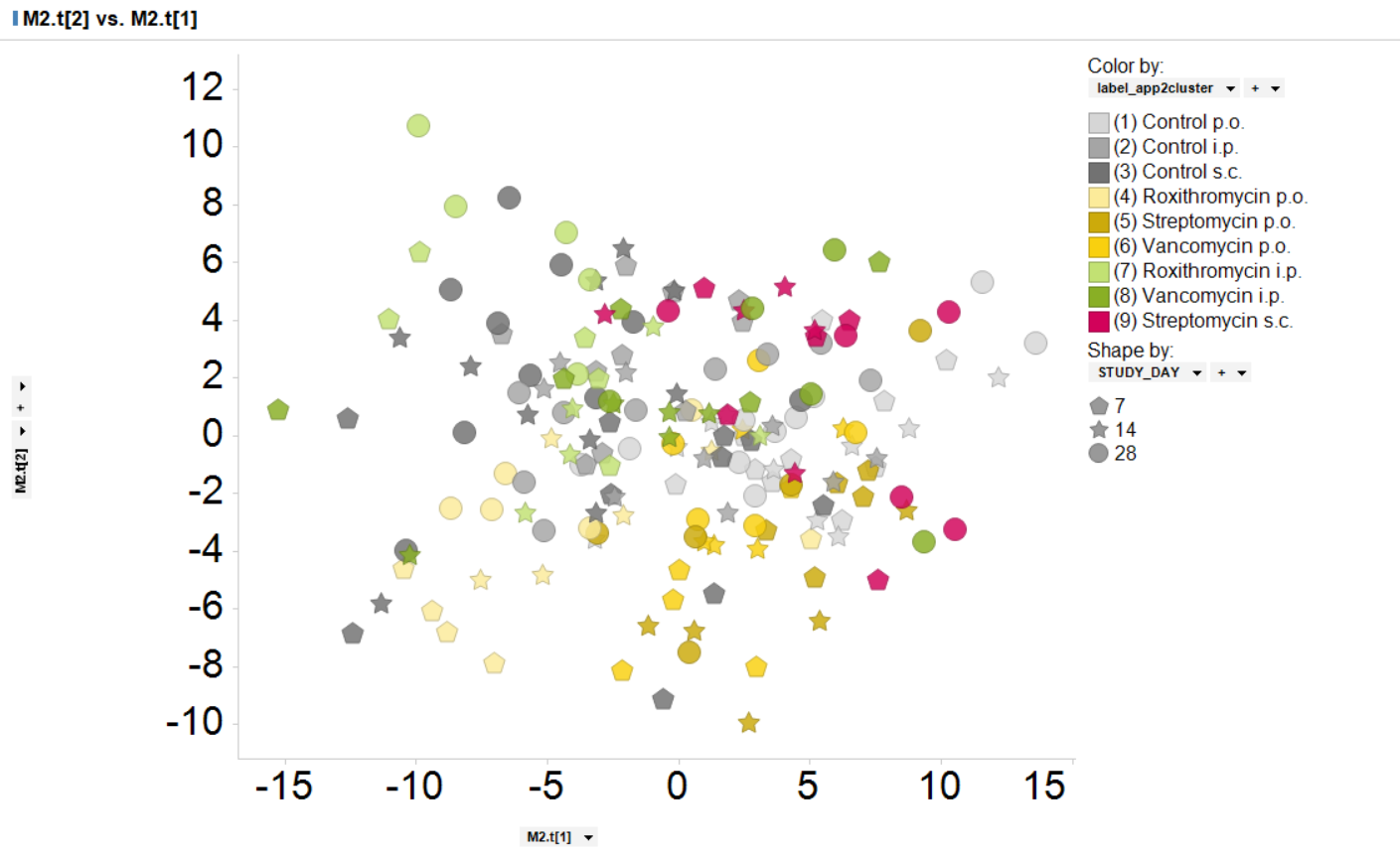 | 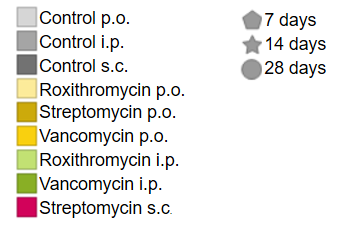 |
| --- | --- | --- |
| b) | p1  p2 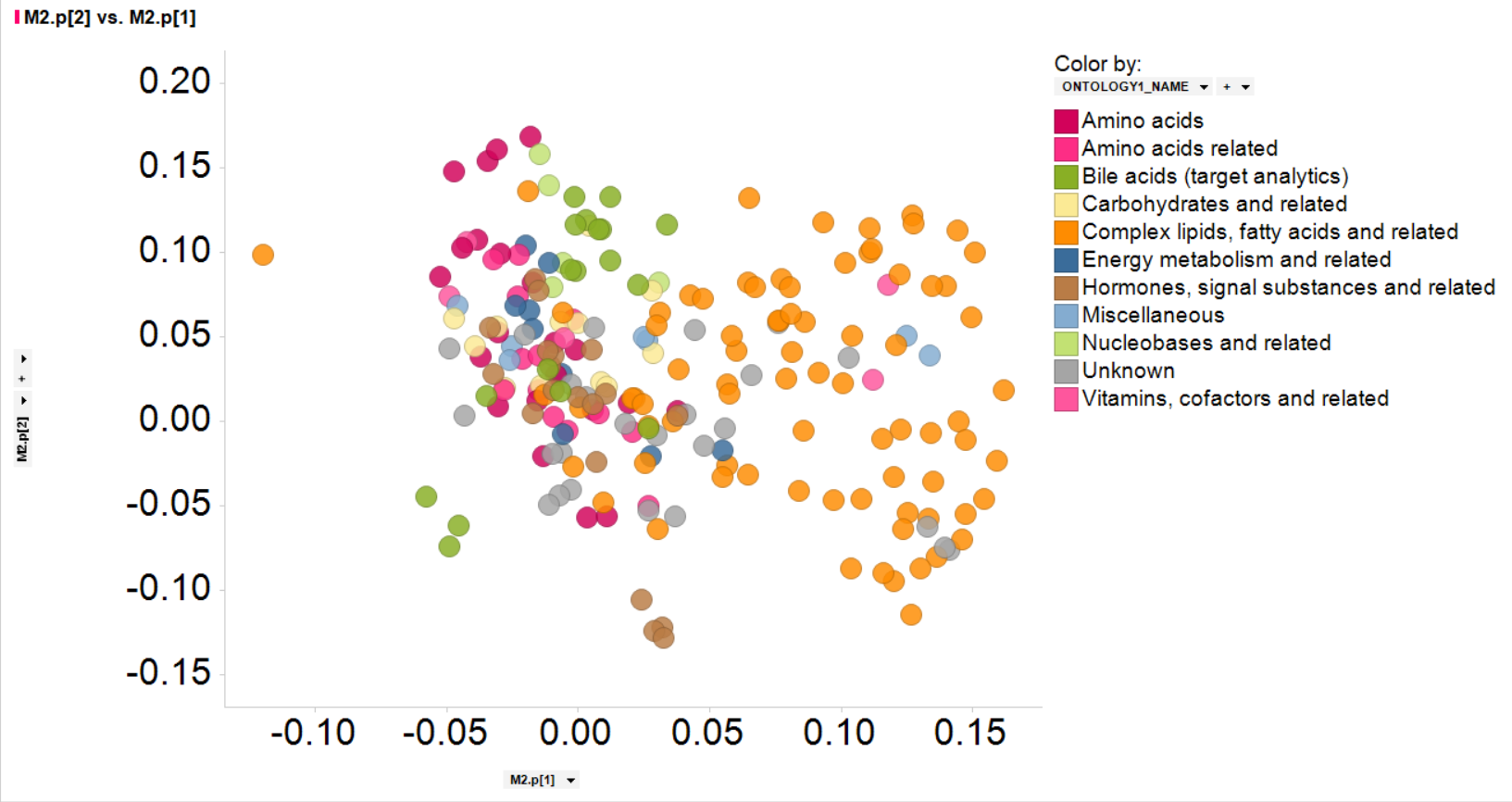 | 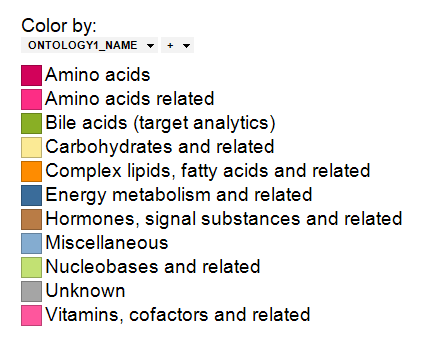 |

**Figure A1.** PCA of treatment-effect of vancomycin, streptomycin and roxithromycin on the plasma metabolome of male rats. a) scores plot. Every dot represents a single plasma sample b) loading plot. Every dot represents a single metabolite. Rats were either orally or parenterally dosed with vancomycin, streptomycin and roxithromycin (N=5 per group). The control group received no treatment, the gavage control group received 0.5% CMC in drinking water and the intraperitoneal and subcutaneous control group were injected with a saline solution (N=10 per group). Blood samples were taken on study days 7, 14 and 28 after overnight fasting and are visualized by different point shapes. PC principle component; p loading for principal component. P.o. per os (by mouth); s.c. subcutaneous; i.p. intraperitoneal

| a)  po1 | 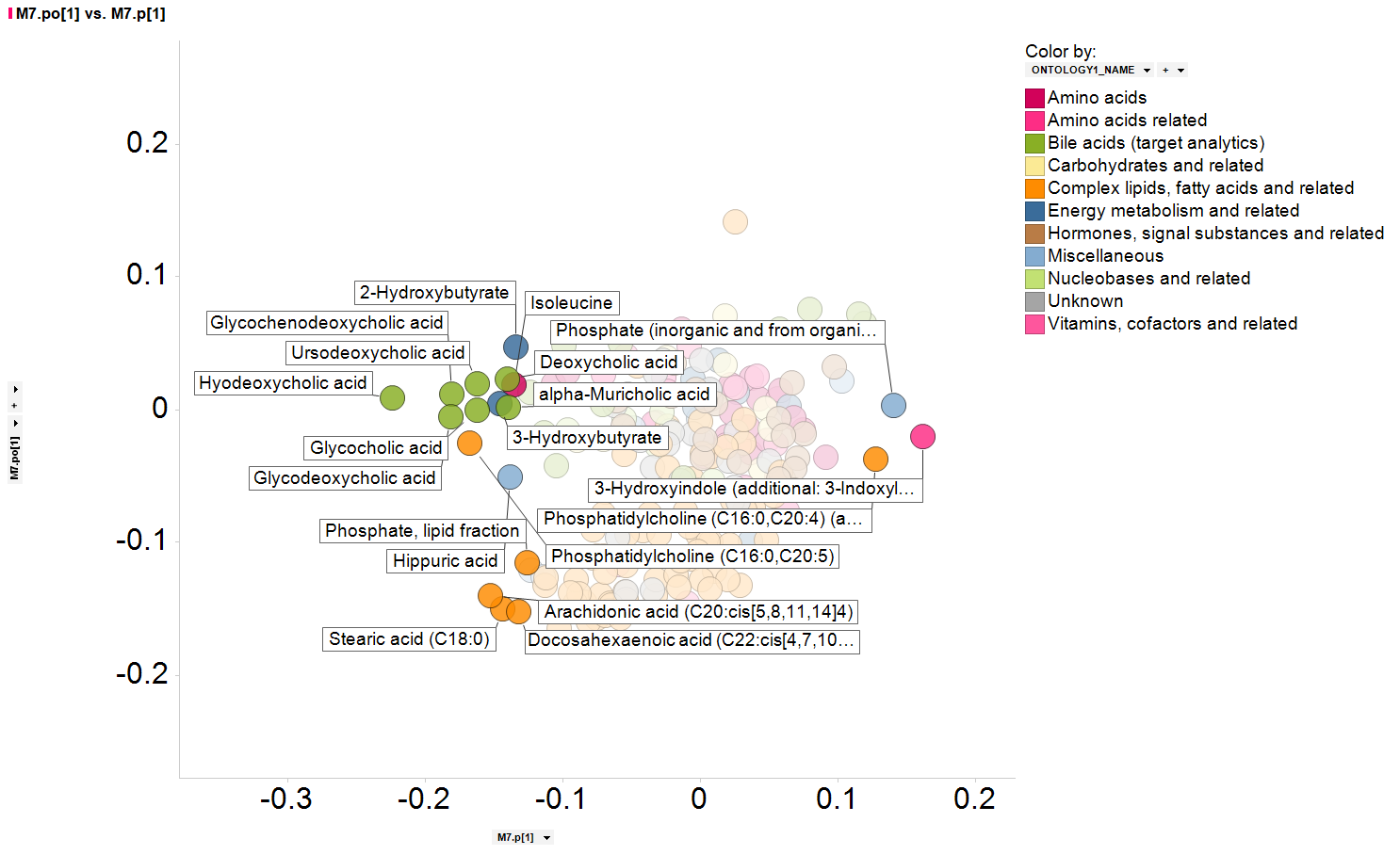 |
| --- | --- |
| b)  po1 | 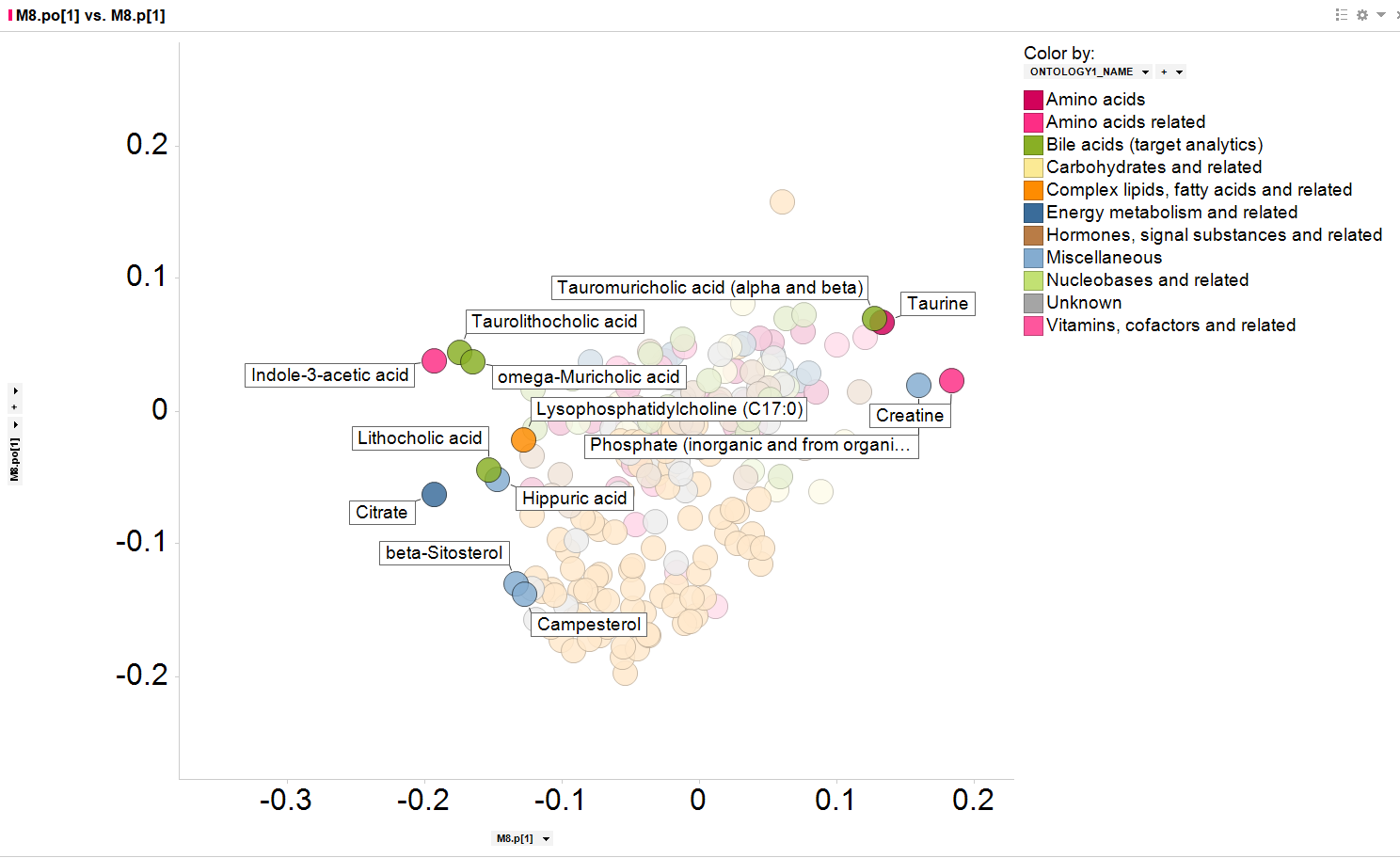 |
| c)  po1 | 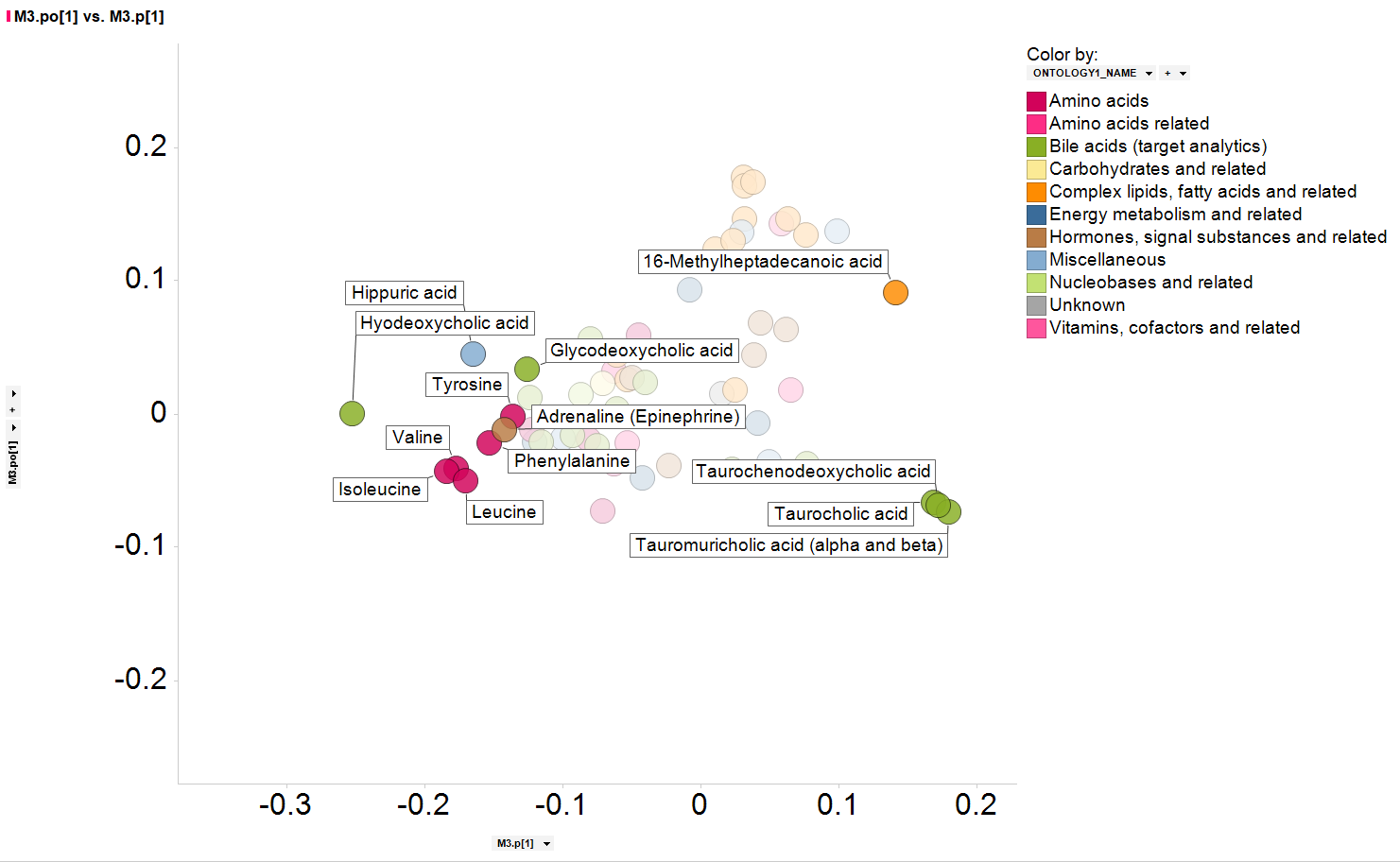 |
| d)  po1 | 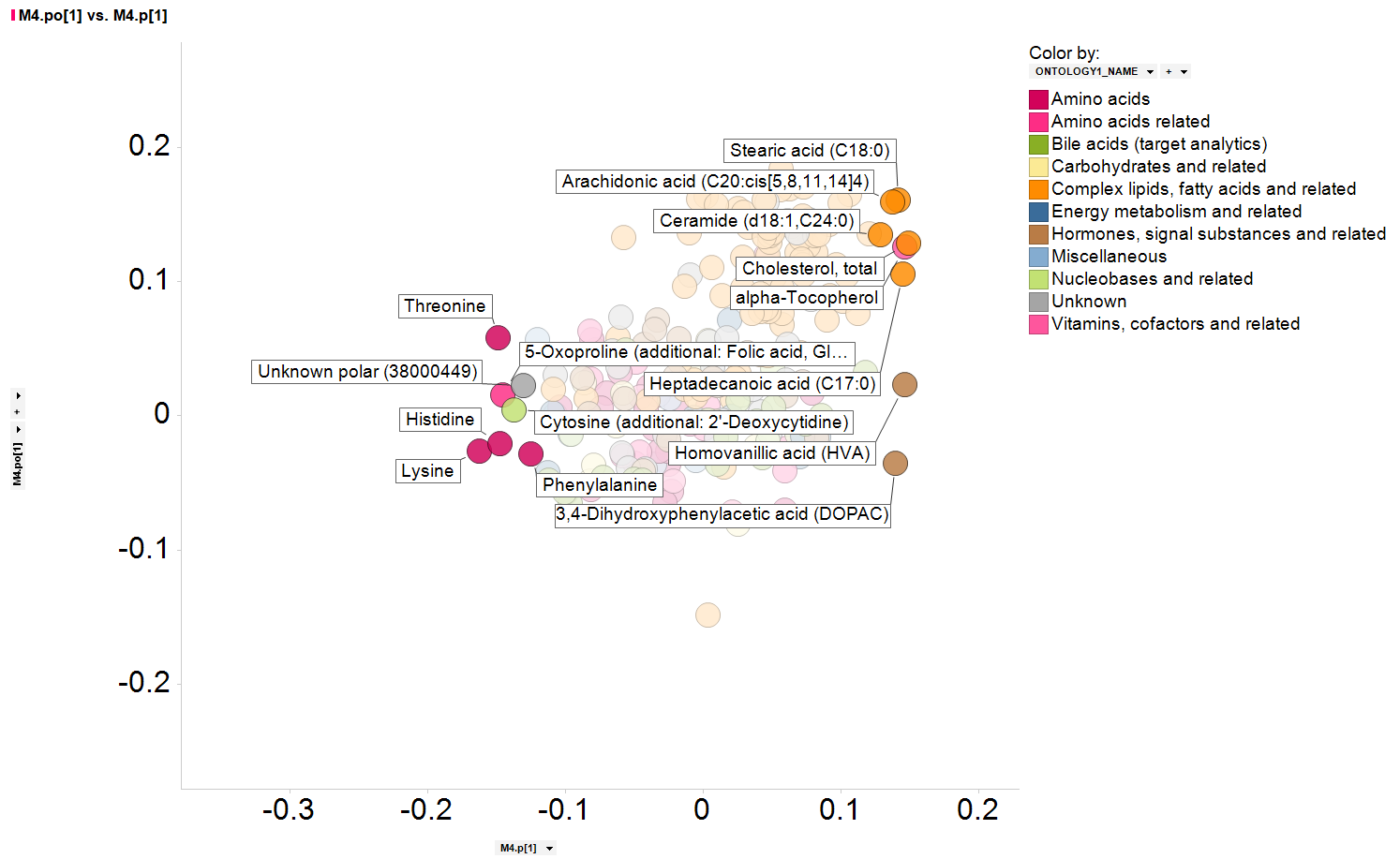 |
| e)  po1 | 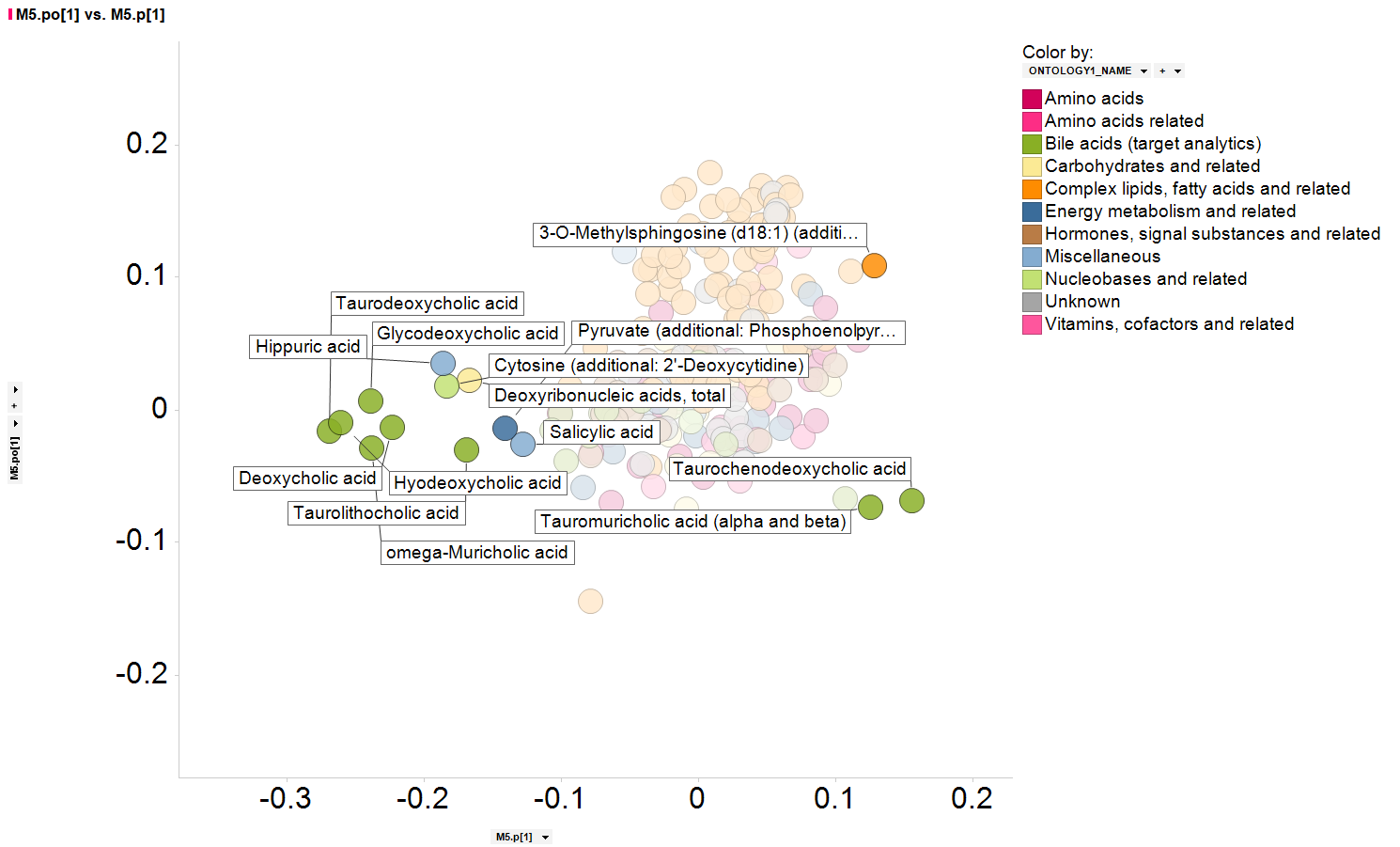 |
| f)  po1 | 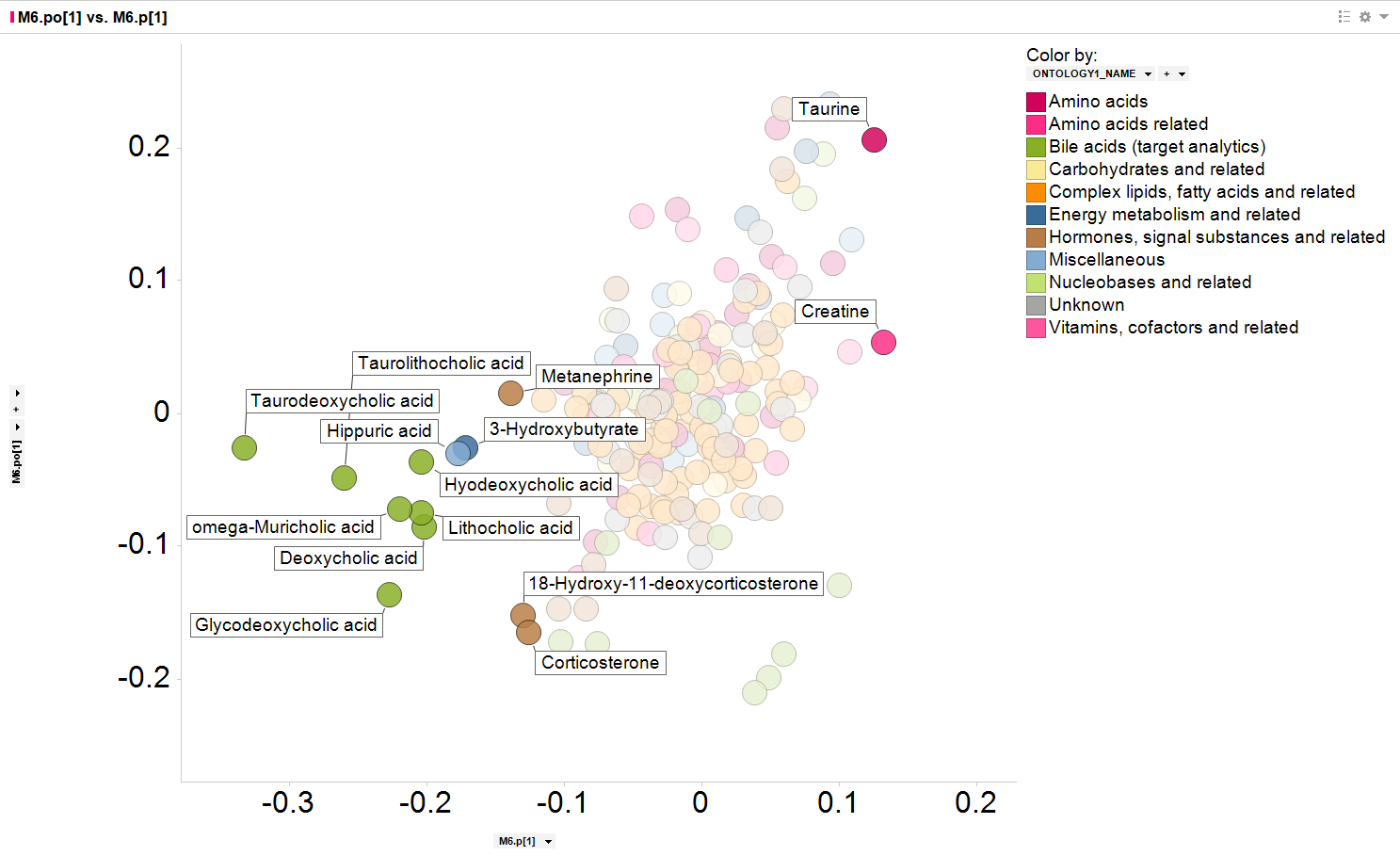 |
|  | pp1 |


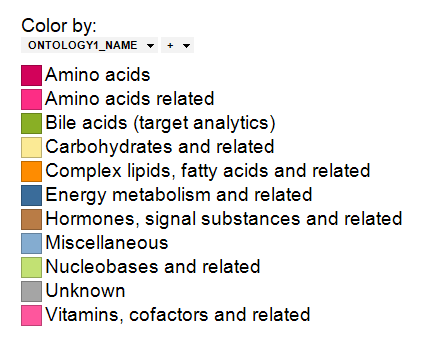


**Figure A2.** O-PLS-DA loading plots. Individual models were generated comparing samples from each antibiotic treatment and application route to all controls. Each dot represents a single metabolite. a) roxithromycin p.o. b) roxithromycin i.p. c) streptomcyin p.o. d) streptomycin s.c. e) vancomycin p.o. f) vancomycin i.p. pp1 loadings for first predictive component; po1 loadings for first orthogonal component; p.o. per os (by mouth); i.p. intraperitoneal; s.c. subcutaneous.
